# Supplementary material for: Adults on pre-exposure prophylaxis (tenofovir-emtricitabine) have faster clearance of anti-HIV monoclonal antibody VRC01
Source: Nat Commun. 2023 Nov 28;14:7813. doi: 10.1038/s41467-023-43399-5 (PMC10684488; doi:10.1038/s41467-023-43399-5)
Supplement: Supplementary file 1 — Supplementary Information [file 41467_2023_43399_MOESM1_ESM.pdf]

Supplementary Information for "**Adults on Pre-Exposure Prophylaxis (tenofovir-emtricitabine) Have Faster Clearance of Anti-HIV Monoclonal Antibody VRC01**"

Methods

DBS Assay

The methods described here are specific to testing for two pre-exposure prophylaxis (PrEP) drug anabolites, intraerythrocytic TFV-DP (tenofovir diphosphate) and FTC-TP (emtricitabine diphosphate).[1,2] TFV-DP is the active metabolite exists within cells after TDF is metabolized, and is responsible for inhibiting viral replication within infected cells; FTC-TP is the active intracellular form generated after FTC is metabolized within cells, and contributes to the overall inhibition of HIV replication via incorporation into viral DNA.

For drug concentration testing, 25 ul of blood from EDTA tubes is spotted five times onto 903 Protein Saver Cards (Whatman/GE Healthcare, Piscataway, NJ) (125 ul blood used in total). After spotting, the cards are dried for at least 2 hours and then placed in plastic bags and stored in a sample box with desiccant and humidity indicators. For short term storage, room temperature (<5 days) or 4C may be used; for longer term storage, -20C and -80C have acceptable stability up to 5 years (reference 6.2, validation report).

For the extraction of analytes from dried blood spot (DBS), a 3-mm diameter disk is punched from the blood spot on the card, using a micropuncher. A punch from a clean Protein Saver Card is performed in between each DBS sample to avoid analyte contamination from the previous DBS punch. The disk is placed in a microcentrifuge tube with 500 ul of 70:30 methanol–water solution. Following extraction, the lysed cellular matrix is subjected to solid phase extraction procedures to prepare the sample for liquid-chromatograpy tandem mass spectrometry. The validated quantifiable linear range for TFV-DP is 25–2000 fmol/sample and that for FTC-TP is 0.1–200 pmol/sample for a 3-mm punch. Because 400ul of the 500ul are assayed (comprising the "sample"), the lower limit of quantification of TFV-DP for a 3mm punch is 31.25 fmol/punch and that for FTC-TP is 0.125 pmol/punch. Stable labeled isotopic internal standards are used to ensure accuracy and precision in various cell matrices.

TFV-DP accumulates appreciably in red blood cells with repeated daily doses and has a long half-life of 17 days in this cellular compartment. Therefore, measurement of TFV-DP in DBS is useful to assess cumulative patterns of adherence over longer periods, and provides valuable insight into both dosing recency and cumulative doses from variable adherence patterns.[3,4] In all subsequent analyses, we defined PrEP use status based on TFV-DP concentration in DBS because the decay kinetics and levels of effective use have been reasonably well established for TFV-DP.

Quantification of VRC Serum Concentrations in the Presence and Absence of TFV/FTC

Emtricitabine (Sigma; PHR2120), TFV (Sigma; PHR1957) and a 2:3 mixture of TFV-DP were diluted in assay diluent to the indicated concentrations and spiked with VRC01. Concentrations tested were assumed to be at least 4 times greater than a theoretical Cmax.[5] Samples were tested in the ELISA format in duplicate at a single dilution level. Average optical density value for each condition and % concentration recovery relative to the VRC01 spike in assay diluent are shown in Supplementary Table 8. All tested conditions showed spike-in concentration recovery within 70-130% of expected.

Supplementary Figure 1a-c. Individual-level baseline markers in PrEP users (n=24) and non-PrEP users (n=24). a) Serum inflammatory marker levels, b) serum ALT and creatinine clearance levels, and c) serum intestinal permeability I-FABP and LBP levels. The mid-line of the box denotes the median and the ends of the box denote the 25th and 75th percentiles. The whiskers at the top and bottom of the box extend to the most extreme data points that are no more than 1.5 times the interquartile range (i.e., height of the box) or if no value meets this criterion, to the data extremes. Open circles indicate individuals who initiated PrEP post-enrollment; stars indicate individuals who initiated PrEP prior to enrollment.

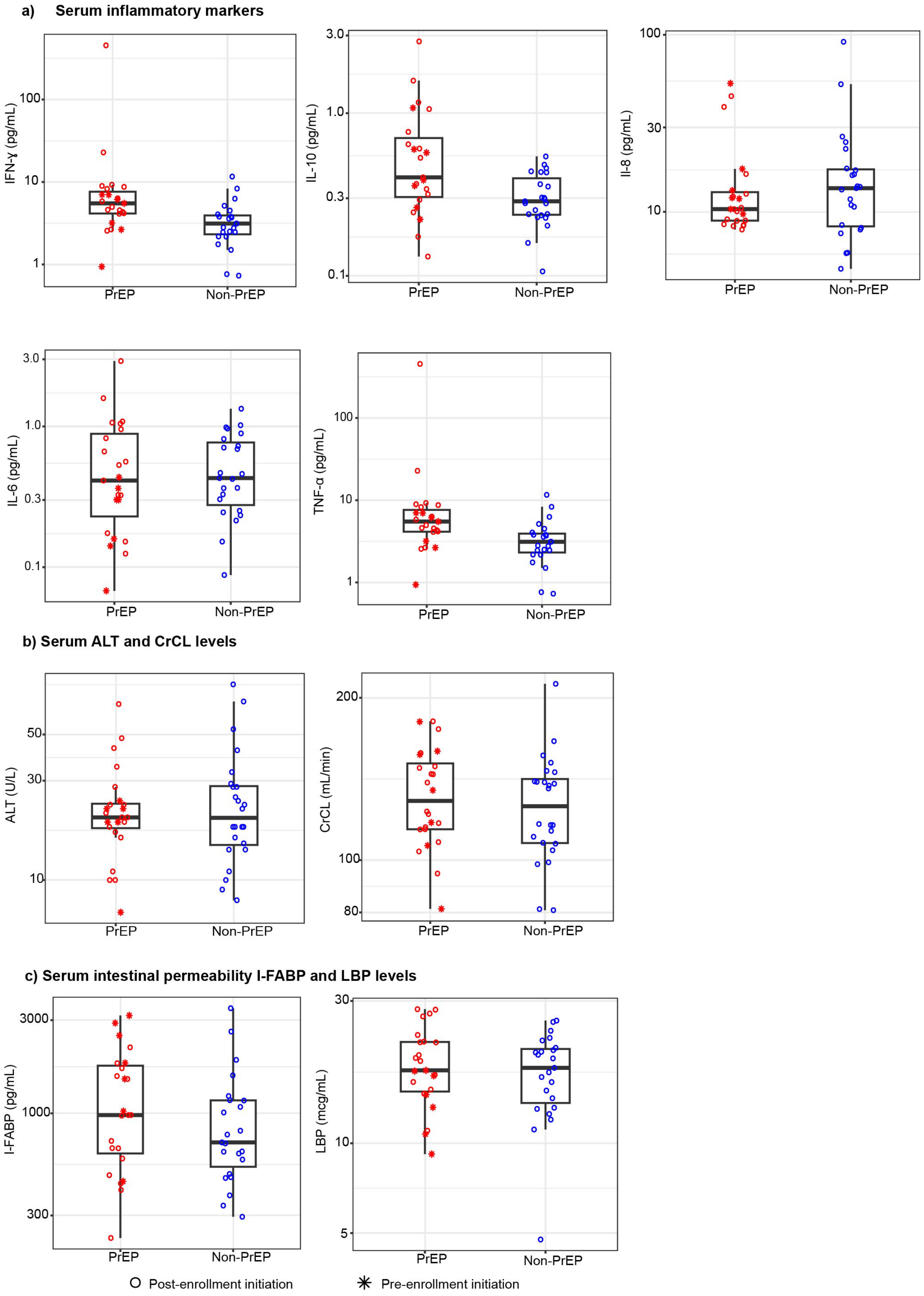
a) Serum inflammatory markers
IFN-γ (pg/mL)
100
10
1
PrEP
Non-PrEP
IL-10 (pg/mL)
3.0
1.0
0.3
0.1
PrEP
Non-PrEP
Il-8 (pg/mL)
100
30
10
PrEP
Non-PrEP
IL-6 (pg/mL)
3.0
1.0
0.3
0.1
PrEP
Non-PrEP
TNF-α (pg/mL)
100
10
1
PrEP
Non-PrEP
b) Serum ALT and CrCL levels
ALT (U/L)
50
30
10
PrEP
Non-PrEP
CrCL (mL/min)
200
100
80
PrEP
Non-PrEP
c) Serum intestinal permeability I-FABP and LBP levels
I-FABP (pg/mL)
3000
1000
300
PrEP
Non-PrEP
LBP (mcg/mL)
30
10
5
PrEP
Non-PrEP
Post-enrollment initiation
Pre-enrollment initiation

Supplementary Figure 2. Correlations between baseline serum intestinal permeability I-FABP and LBP levels, and inflammatory marker levels. The pairwise scatterplots are shown in the lower diagonal for each pair of markers. The histograms of each marker level are shown in the diagonal. The Spearman correlation coefficients are shown in the upper diagonal with font sizes proportional to the magnitude of the correlation coefficients.

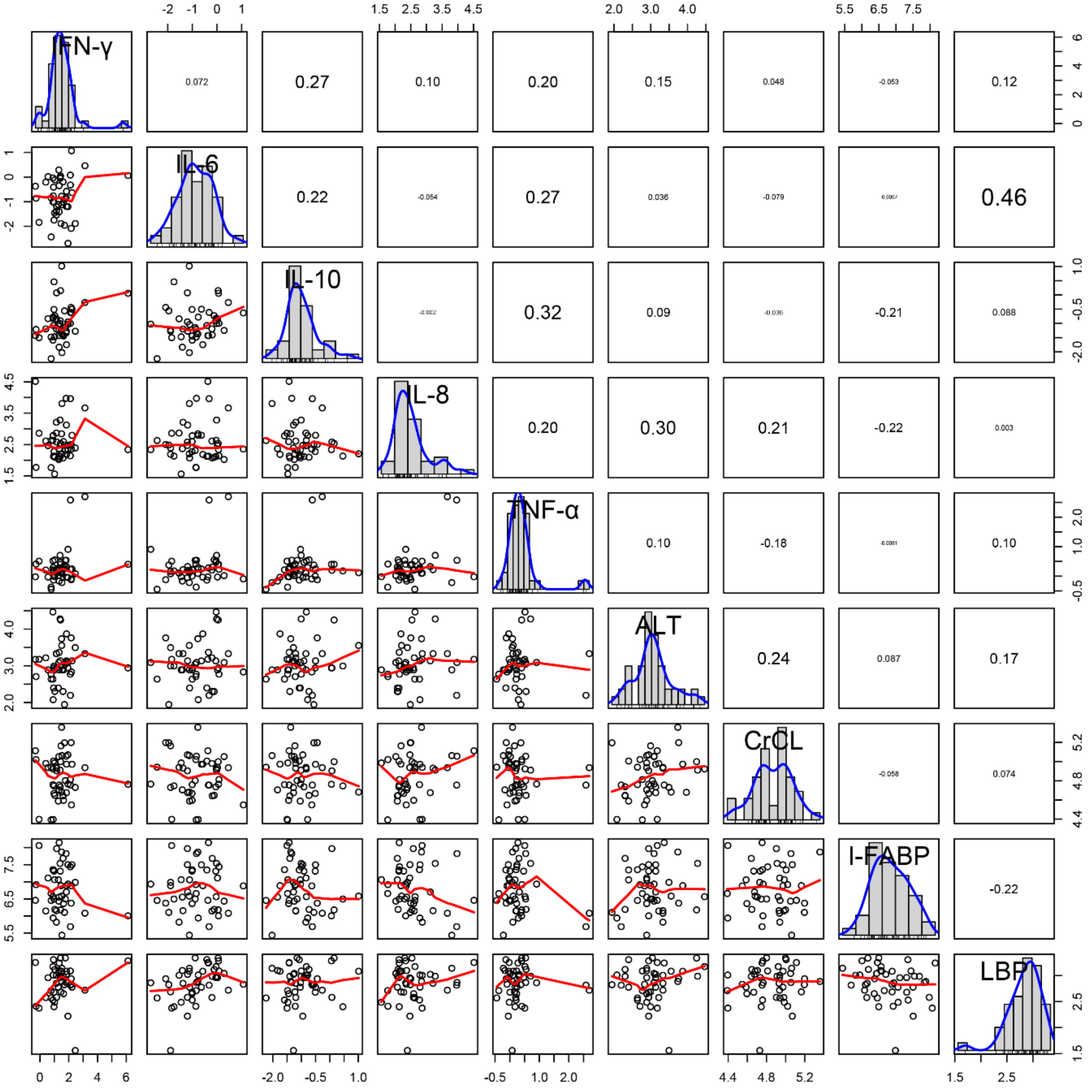

Supplementary Figure 3a-b. Uniform Manifold Approximation and Projection (UMAP) plot showing how separable, or lack of, PrEP users and non-PrEP users are with respect to all baseline characteristics presented in Table 1. a) includes data from all non-PrEP users (n=24) and PrEP users who initiated PrEP after study enrolment (n=17); b) includes data from all non-PrEP users (n=24) and all PrEP users (n=24).

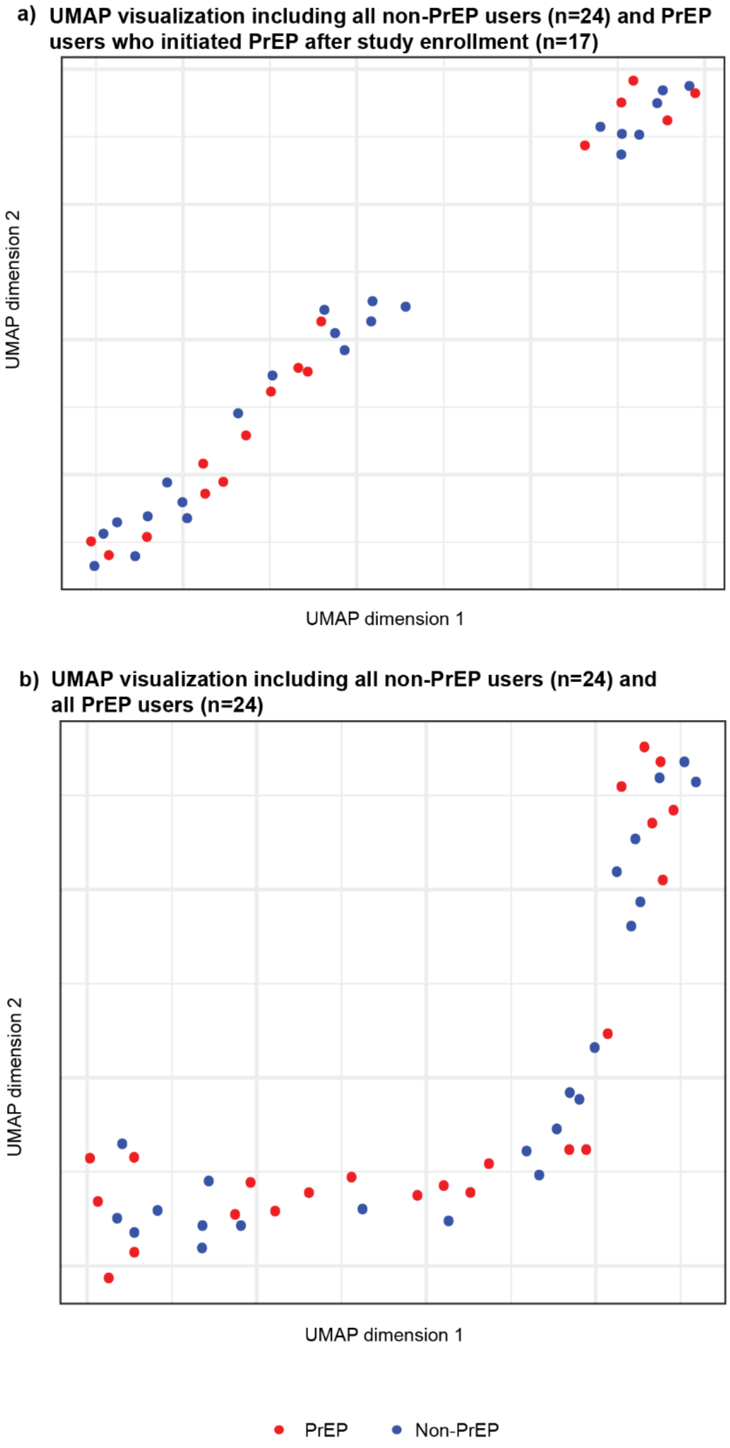

Supplementary Figure 4a-e. Individual-level estimated PK parameters in PrEP users and non-PrEP users. a) Clearance (CL, L/day); b) volume of the peripheral compartment (Vp, L); c) distribution half-life (day); d) elimination half-life (day), and e) steady state area under the curve (AUC, day/mL). The mid-line of the box denotes the median and the ends of the box denote the 25th and 75th percentiles. The whiskers at the top and bottom of the box extend to the most extreme data points that are no more than 1.5 times the interquartile range (i.e., height of the box) or if no value meets this criterion, to the data extremes. Open circles indicate individuals who initiated PrEP post-enrollment; stars indicate individuals who initiated PrEP prior to enrollment.

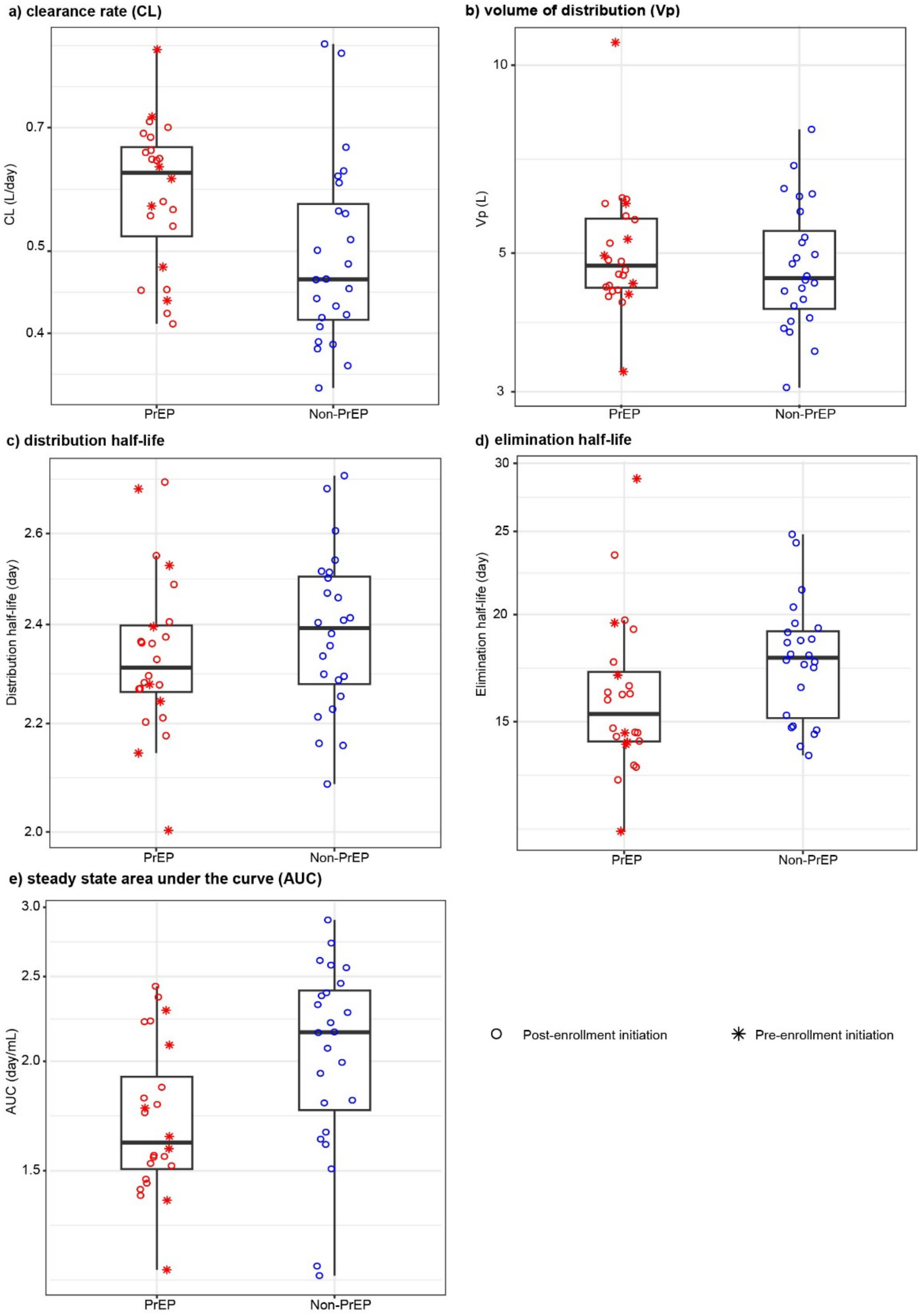

a) clearance rate (CL)
CL (L/day)
0.7
0.5
0.4
PrEP
Non-PrEP
b) volume of distribution (Vp)
Vp (L)
10
5
3
PrEP
Non-PrEP
c) distribution half-life
Distribution half-life (day)
2.6
2.4
2.2
2.0
PrEP
Non-PrEP
d) elimination half-life
Elimination half-life (day)
30
25
20
15
PrEP
Non-PrEP
e) steady state area under the curve (AUC)
AUC (day/mL)
3.0
2.5
2.0
1.5
PrEP
Non-PrEP
Post-enrollment initiation
Pre-enrollment initiation

Supplementary Figure 5. PK parameter estimates (CL, L/day) from the base popPK model vs. normalized covariate. For continuous covariates, the normalized value (scaled by median) was used, and a loess smooth line was used to help visualize trends. For discrete variables, boxplots were used for each category. Spearman correlation coefficients (*r*) and p-values for testing non-zero coefficients are included.

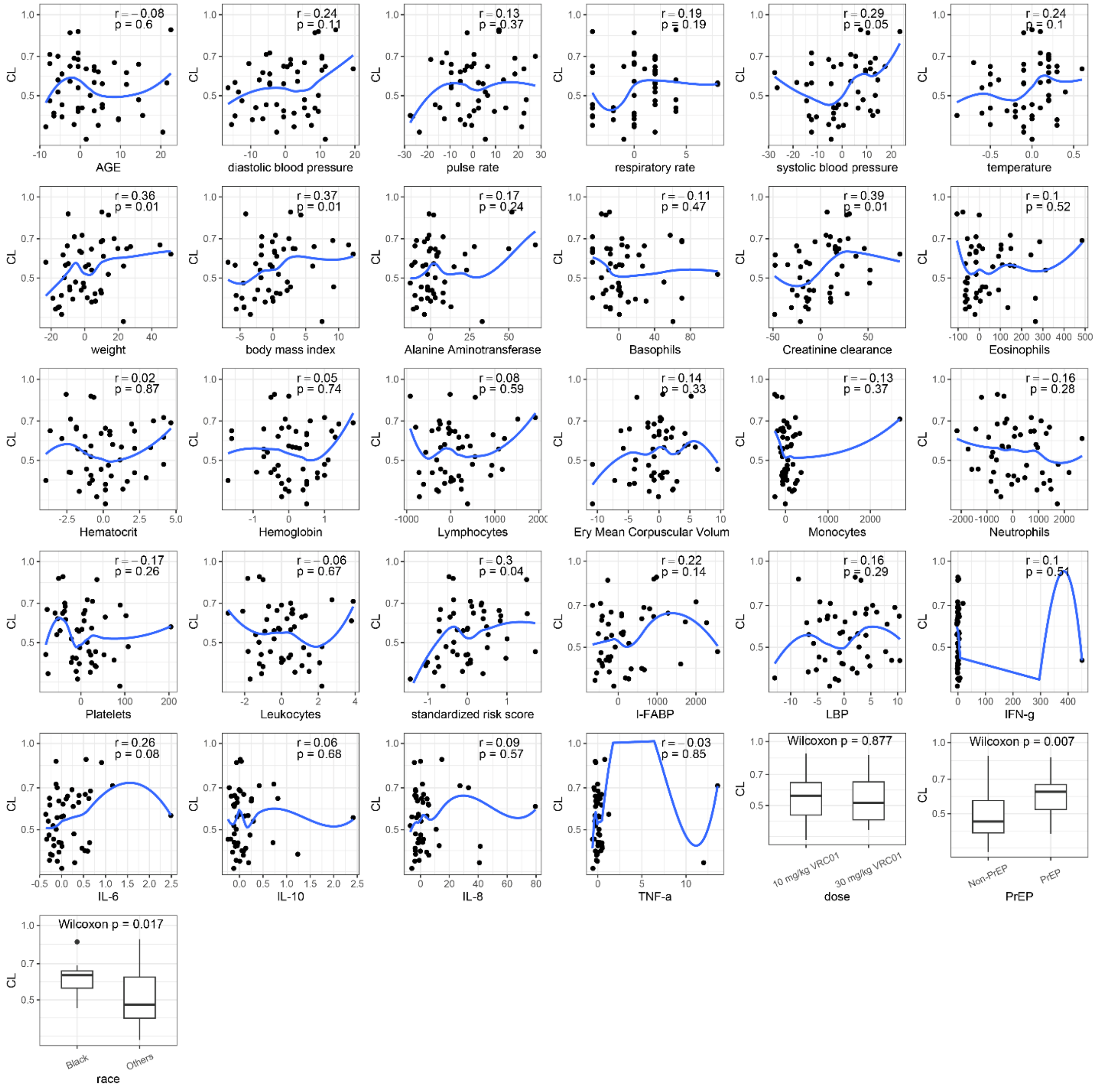

Supplementary Figure 6. PK parameter estimates (Vp, L) from the base model vs. normalized covariate. For continuous covariates, the normalized value (scaled by median) was used, and a loess smooth line was used to help visualize trends. For discrete variables, boxplots were used for each category. Spearman correlation coefficients (*r*) and p-values testing for non-zero coefficients are included.

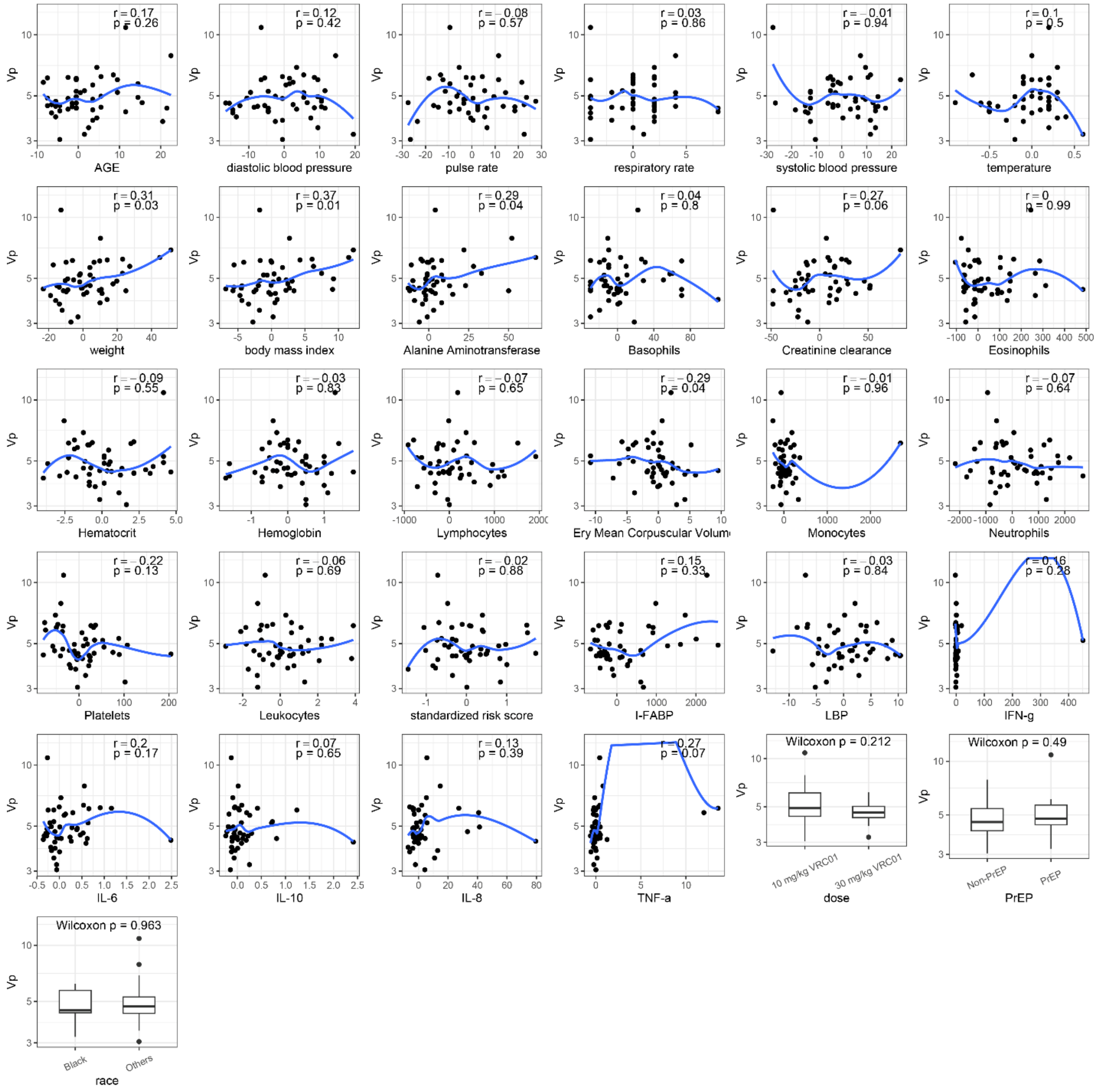

Supplementary Figure 7. **Diagnostic plots of the final time-dependent PrEP popPK model with individual-level VRC01 serum concentrations over time by dose group (n=48)**. Black lines show the observed VRC01 serum concentrations; blue lines show the predicted VRC01 serum concentrations accounting for individual-specific variability in PK models from the popPK model, and red lines show the predicted VRC01 serum concentration without accounting for individual-specific variability in PK parameters.

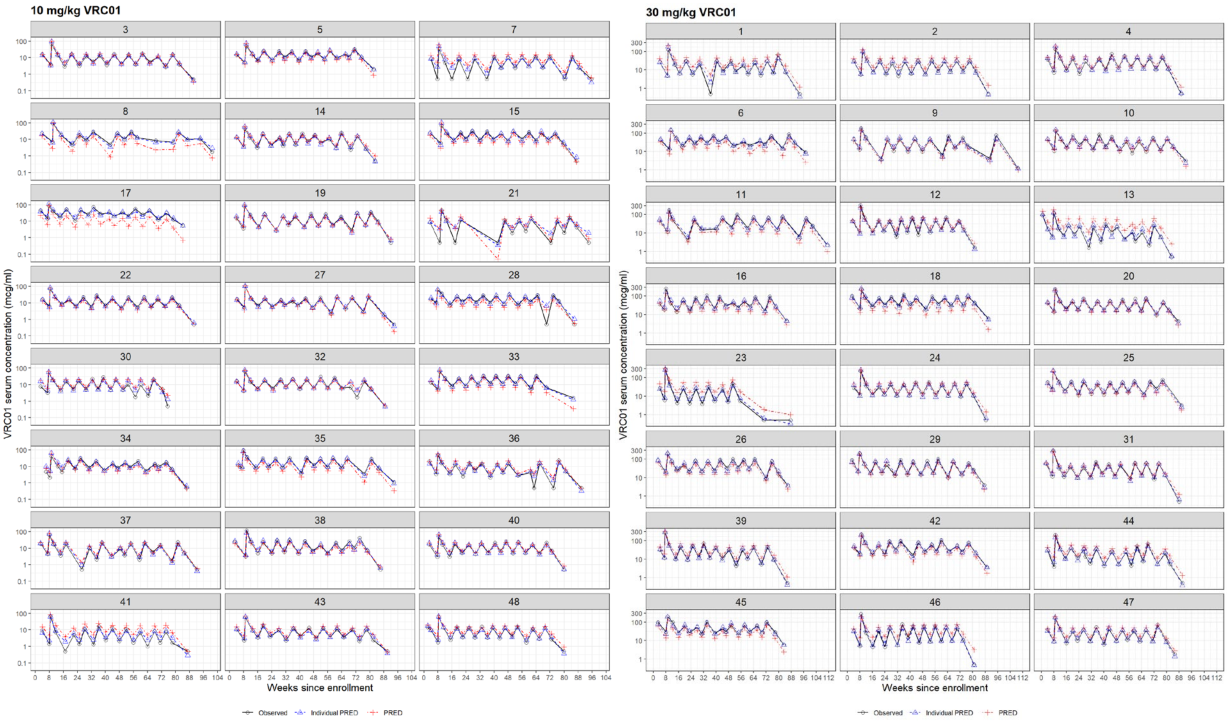

Supplementary Figure 8a-f. Goodness-of-fit model diagnostic plots obtained from the final popPK model with time-dependent PrEP use (N=48). (a,b) Observed vs. individual-predicted concentration with an identity line [(a) linear scale; (b) log scale], c) Individual weighted residual (IWRES) residual errors vs. individual predictions, d) Individual weighted residual (IWRES) residual errors vs. time, e) Distribution of inter-individual random effects, f) Scatter visual predictive check (VPC). Data from multiple time-points of a participant are individually displayed, not aggregated.

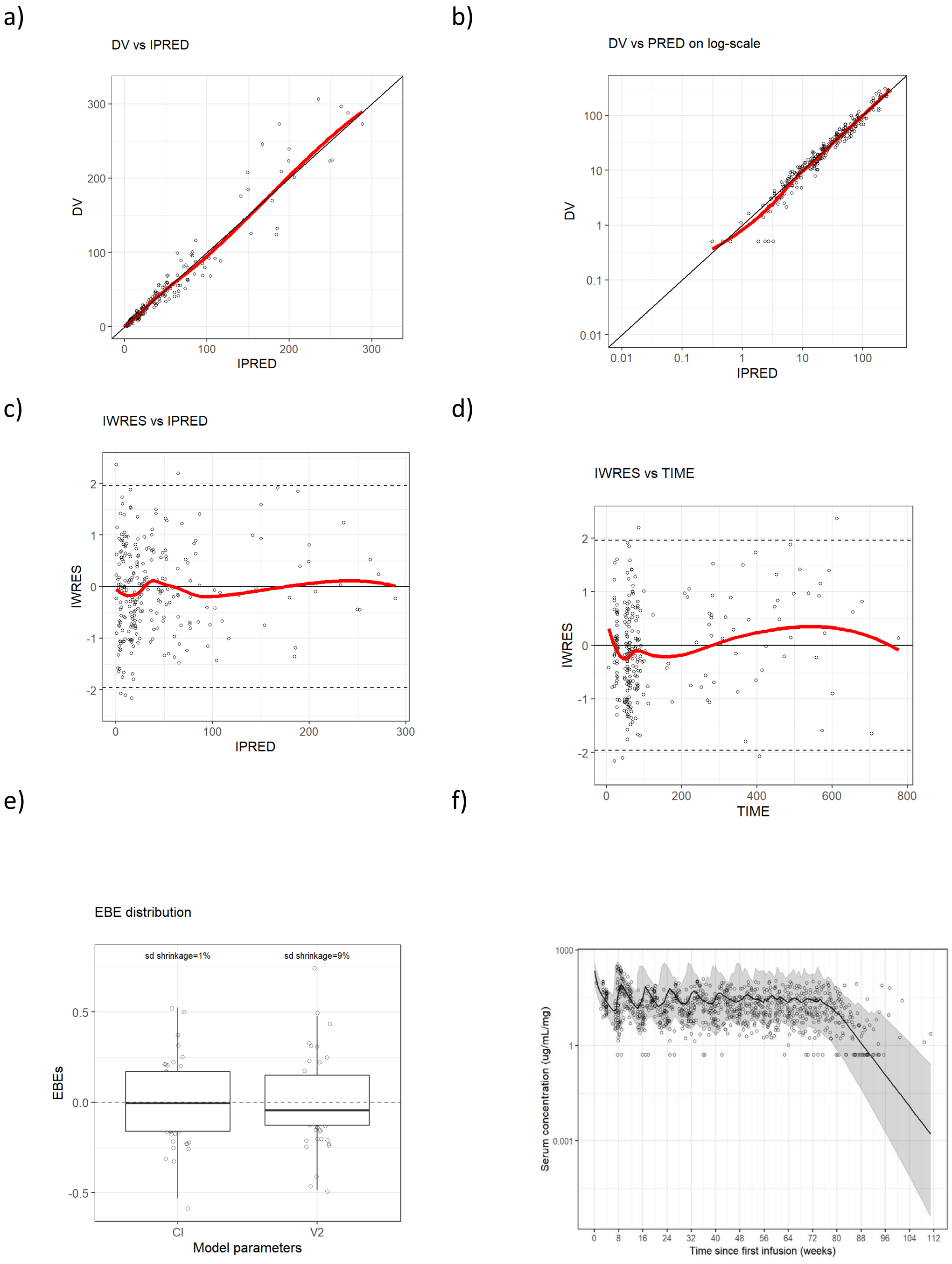

Supplementary Figure 9. Individual-level Serum Cystatin C Level-based Glomerular Filtration Rate (GFR). GRF is calculated based on the CKD-EPI (Chronic Kidney Disease Epidemiology Collaboration) equation: GFR (mL/min/1.73 m^2) = 127.7 × (Cystatin C / 0.8)^(-1.328) × (Age / 70)^(-0.076) × 0.996^Male. The "early visit on PrEP" time-point was defined as the earliest study visit after the first evidence of PrEP use based on self-report and DBS. For the 17 PrEP users who had no evidence of PrEP use prior to enrollment, this "early visit on PrEP" was approximately 4 weeks after the first evidence of PrEP use during the AMP study; for the 7 PrEP users who had evidence of PrEP use prior to enrollment, this "early visit on PrEP" was the enrollment visit. The "last visit on PrEP" visit was the last study visit with evidence of PrEP use, typically the Week 72 visit for the 10th infusion, and a median of 439 days (IQR 233-546 days) since the self-reported date of PrEP uptake. The mid-line of the box denotes the median and the ends of the box denote the 25th and 75th percentiles. The whiskers at the top and bottom of the box extend to the most extreme data points that are no more than 1.5 times the interquartile range (i.e., height of the box) or if no value meets this criterion, to the data extremes.

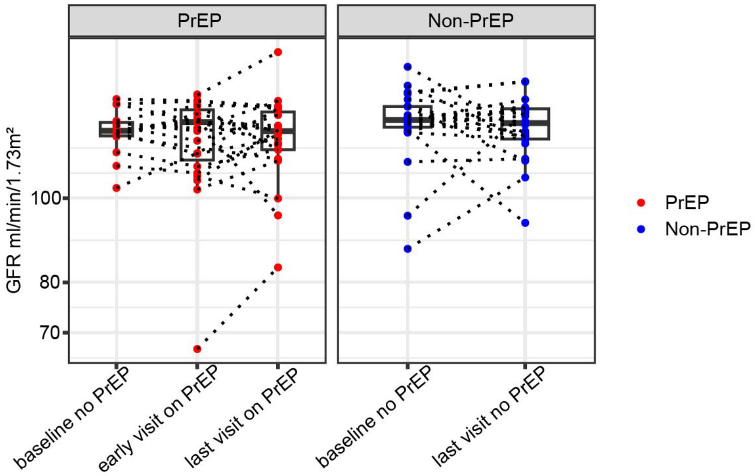

Supplementary Figure 10. Correlation between I-FABP Levels and TFV-DP levels at the Last Study Visit on PrEP among PrEP Users. The Spearman correlation coefficient and corresponding p-value for testing a non-zero correlation is included. A linear regression line is also included.

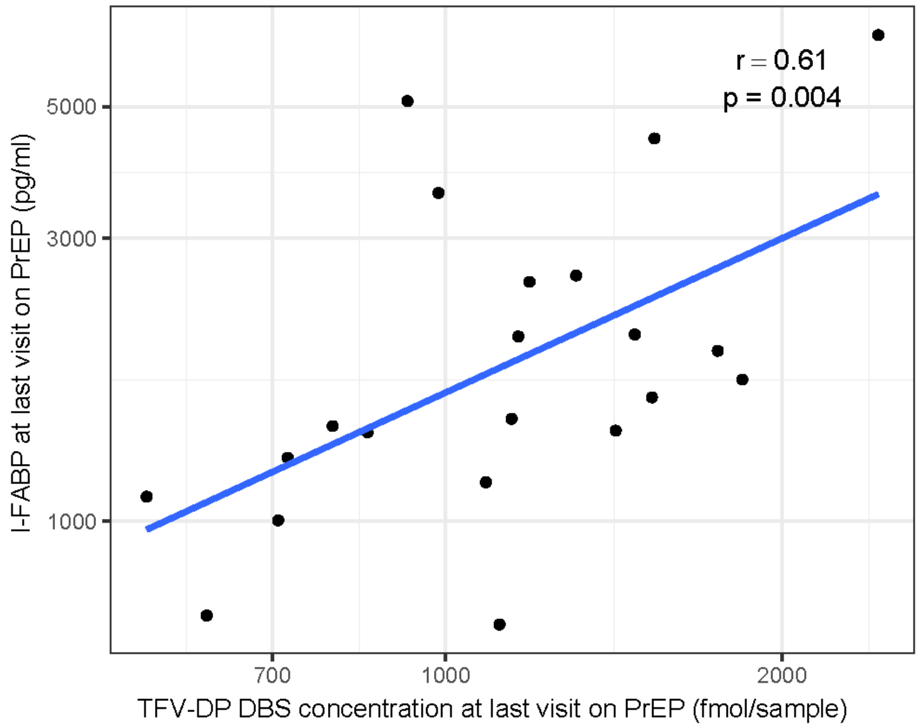

Supplementary Figure 11. Schematic of the performed analyses

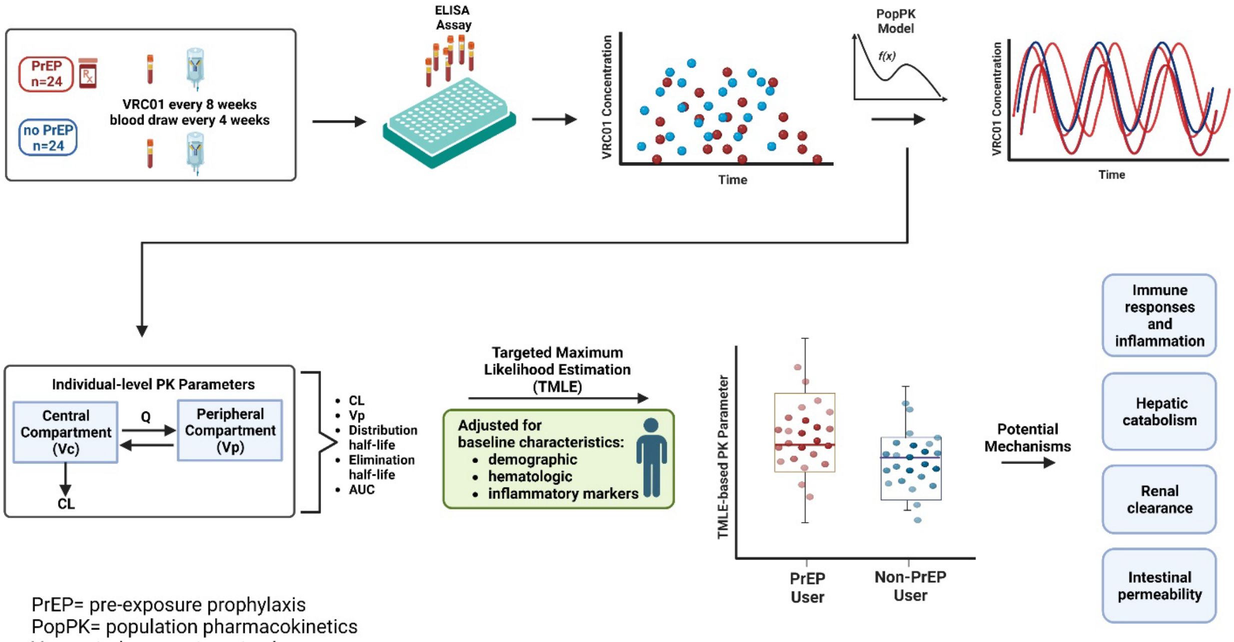

PrEP= pre-exposure prophylaxis
PopPK= population pharmacokinetics
Vc=central compartment volume
CL=clearance rate
Q= distribution and redistribution rate
Vp= peripheral volume of distribution
AUC= steady state dose-normalized area-under-the-curve

Created with BioRender.com

Supplementary Table 1. Baseline characteristics of study participants by PrEP user status and time of PrEP initiation. A participant was defined as a PrEP user if they met all of the following criteria during the study: 1) accessed the PrEP referral program on at least one occasion per self-report, 2) self-reported intermittent or continuous PrEP use, and 3) confirmation of ≥3 positive tenofovir detection tests out of DBS samples collected at infusion visits. A participant was defined as a non-PrEP user if they did not self-report any PrEP use and had no DBS samples tested positive for tenofovir.

Covariates	Categories	Non-PrEP Users N=24*	PrEP Users N=24*	PrEP use after AMP enrollment N=17*	PrEP use prior to AMP enrollment N=7
		N (%)			
**VRC01 Dose (mg/kg)**	10 mg/kg	12 (50%)	12 (50%)	10 (58.8%)	2 (28.6%)
	30 mg/kg	12 (50%)	12 (50%)	7 (41.2%)	5 (71.4%)
Demographics					
**Sex assigned at birth**	Male	23 (95.8%)	24 (100%)	17 (100%)	7 (100%)
	Female	1 (4.2%)	0 (0%)	0 (0%)	0 (0%)
**Race**	Black	6 (25.0%)	7 (29.2%)	6 (35.3%)	1 (14.3%)
	White	15 (62.5%)	11 (45.83%)	7 (41.18%)	4 (57.14%)
	Other	3 (12.5%)	6 (25%)	4 (23.53%)	2 (28.57%)
		Median (Range)			
**Body mass index (kg/m2)**		25.8 (19.7, 37.6)	25.4 (18.6, 34.5)	25.4 (18.6, 34.5)	23.7 (20.9, 26.5)
**Weight (kg)**		75.2 (59.9, 130.2)	80.8 (55.7, 106.1)	83 (55.7, 106.14)	75.93 (66, 85)
**Age (y)**		31 (19, 50)	27 (20, 43)	27 (20, 43)	27 (20, 39)
Vital Signs					
**Diastolic blood pressure (mmHg)**		74 (59, 89)	76 (58, 94)	75 (58, 86)	78 (62, 94)
**Systolic blood pressure (mmHg)**		122 (109, 136)	124 (95, 146)	122 (96, 140)	126 (95, 146)
**Pulse rate (beats/min)**		77 (51, 101)	78 (50, 105)	78 (50, 105)	80 (66, 100)
**Respiratory rate (breaths/min)**		16 (12, 24)	17 (12, 24)	18 (12, 24)	16 (12, 20)
**Temperature (°C)**		36.6 (36.0, 37.1)	36.8 (35.8, 37.3)	36.8 (35.8, 37)	36.9 (36.5, 37.3)
Safety hematological markers					
**Alanine aminotransferase (unites/L)**		20 (8, 87)	20 (7, 70)	20 (10, 70)	19 (7, 24)
**Basophils (cells/mm^3)**		31 (0, 140)	23 (0, 100)	26 (0, 100)	20 (0, 87)
**Creatinine clearance (mL/min)**		126.2 (80.8, 212.3)	128.9 (81.2, 180.9)	123.1 (94.4, 180.9)	134.8 (81.2, 180.6)
**Eosinophils (cells/mm^3)**		101 (38, 380)	120 (10, 600)	120 (53, 600)	100 (10, 359)
**Hematocrit (%)**		43.6 (39.9, 48.0)	44.1 (40.2, 48.5)	44 (40.2, 48.5)	44.2 (43, 48)
**Hemoglobin (g/dl)**		14.6 (12.8, 15.6)	14.5 (12.9, 16.3)	14.5 (12.9, 16.3)	14.5 (14.2, 15.8)
**Lymphocytes (cells/mm^3)**		1932 (1043, 3058)	1800 (920, 3759)	1700 (1118, 3360)	2023 (920, 3759)
**Erythrocyte mean corpuscular volume (fL)**		90.4 (80.0, 100.3)	91 (84.6, 96.7)	90.8 (84.6, 96.7)	92.8 (89.1, 96)
**Monocytes (cells/mm^3)**		455 (211, 740)	485 (260, 3160)	470 (273, 3160)	504 (260, 800)
**Neutrophils (cells/mm^3)**		3192 (1712, 5988)	3370 (1138, 5483)	3398 (1138, 5483)	3342 (2366, 4600)
**Platelets (10^3/mm^3)**		229.5 (148.0, 335.0)	227.5 (169.0, 432.0)	230 (169, 432)	225 (177, 330)
**Leukocytes (10^3/mm^3)**		5.9 (4.2, 9.8)	6 (3.1, 9.9)	6 (3.1, 9.9)	6 (4.2, 8.7)
Inflammatory markers (pg/mL)					
**IFN-γ**		3.1 (0.7, 11.6)	5.5 (0.9, 454.9)	5.4 (2.6, 454.9)	5.5 (0.9, 7.0)
**IL-6**		0.4 (0.1, 1.3)	0.4 (0.1, 2.9)	0.6 (0.1, 2.9)	0.3 (0.1, 0.4)
**IL-8**		13.6 (4.8, 91.3)	10.4 (8.0, 53.2)	9.6 (8.0, 45.1)	12 (9.7, 53.2)
**IL-10**		0.29 (0.11, 0.54)	0.4 (0.1, 2.8)	0.5 (0.1, 2.8)	0.4 (0.2, 1.1)
**TNF-α**		1.2 (0.6, 13.2)	1.3 (0.8, 14.8)	1.2 (0.8, 14.8)	1.5 (1.0, 2.5)
**LBP (ng/mL)**		17.9 (4.8, 25.8)	17.6 (9.2, 28.1)	20.8 (11.0, 28.1)	14.6 (9.2, 17.6)
Intestinal permeability (pg/mL)					
**I-FABP (pg/mL)**		710.1 (295.6, 3453.0	980.2 (230.3, 3169.3)	847.9 (230.3, 2177.5)	1811.3 (448.6, 3169.3)
**LBP (mcg/mL)**		17.9 (4.8, 25.8)	17.6 (9.2, 28.1)	20.8 (11.0, 28.1)	14.6 (9.2, 17.6)
HIV-1 exposure					
**Behavioral risk score**		-0.9 (-2.2, 1.0)	-0.6 (-1.8, 0.8)	-0.6 (-1.8, 0.8)	-0.7 (-1.4, 0.3)

PrEP: pre-exposure prophylaxis; AMP: Antibody mediated prevention

*One non-PrEP user participant and one PrEP user participant who started PrEP after enrollment were missing baseline specimens and hence missing cytokine and permeability marker measurements.

Supplementary Table 2. Number and proportion of participants who received each infusion by dose group.

dose	PrEP	infusion #1	infusion #2	infusion #3	infusion #4	infusion #5	infusion #6	infusion #7	infusion #8	infusion #9	infusion #10
10 mg/kg VRC01	PrEP	12 (100%)	12 (100%)	12 (100%)	11 (91.7%)	11 (91.7%)	12 (100%)	12 (100%)	12 (100%)	12 (100%)	12 (100%)
	Non-PrEP	12 (100%)	12 (100%)	12 (100%)	12 (100%)	12 (100%)	12 (100%)	12 (100%)	12 (100%)	12 (100%)	12 (100%)
30 mg/kg VRC01	PrEP	12 (100%)	12 (100%)	12 (100%)	12 (100%)	12 (100%)	12 (100%)	12 (100%)	12 (100%)	12 (100%)	12 (100%)
	Non-PrEP	12 (100%)	12 (100%)	12 (100%)	12 (100%)	12 (100%)	12 (100%)	12 (100%)	12 (100%)	11 (91.7%)	12 (100%)

Supplementary Table 3. Estimates of PK parameters from the base popPK model. The base model is based on a two-compartment PK model parametrized in terms of CL, Vc, Q and Vp, with random effects for CL and Vp without including any covariates to explain the inter-individual variability in the PK parameters.

**Parameter**	**Description**	**Estimate**	**95% CI**	**%RSE**
**Fixed Effects**				
CL (L/day)	Clearance	0.54	(0.49, 0.58)	4.31
Vc (L)	Central volume	5.19	(4.69, 5.70)	4.96
Q (L/day)	Inter-compartmental clearance	0.56	(0.36, 0.77)	18.40
Vp (L)	Peripheral volume	4.85	(3.90, 5.81)	10.07
**Random Effects**				
CL (L/day)	SD, clearance	0.24	(0.19, 0.29)	10.89
Vp (L)	SD, peripheral volume	0.25	(0.18, 0.32)	13.81
**Error Model Parameters**				
Proportional error	SE	0.24	(0.23, 0.26)	2.43

RSE: relative standard error; CI: confidence interval: SE: standard error

Supplementary Table 4. Sensitivity analysis of covariate-adjusted comparisons of PK features between non-PrEP users (n=24) and PrEP users (n=23) via TMLE. All comparisons were adjusted for age, body weight, race, risk score, creatinine clearance, IFN-gamma, and IL-10 levels. Data from one PrEP user was removed from the TMLE analysis due to unstable PK parameter estimates. All TMLE estimation results of means were averaged over 20 runs with a fixed random seed on top of the 10-fold cross-validation estimation procedure to ensure stability of the estimates. A bootstrap procedure based on 500 datasets was used to calculate the empirical variances of the estimates for each group and to derive the 95% confidence interval, as well as to test for a non-zero mean difference between the two groups via the Wald test. The Holm method was used to adjust for multiple comparisons of the five PK features.

**PK feature**	**PrEP User Status**	**Mean[a] (95% CI)[b]**	**2-sided raw p-value[b]**
Clearance [CL (L/day)]	PrEP	0.60 (0.54, 0.66)	**0.003**
	Non-PrEP	0.53 (0.46, 0.59)	
	Difference	0.08 (0.03, 0.13)	
Volume of the peripheral compartment [Vp (L)]	PrEP	4.86 (2.08, 7.65)	0.33
	Non-PrEP	5.00 (2.28, 7.73)	
	Difference	-0.14 (-0.41, 0.14)	
Distribution half-life (day)	PrEP	2.32 (-3.41, 8.05)	0.80
	Non-PrEP	2.38 (-3.56, 8.31)	
	Difference	-0.05 (-0.46, 0.36)	
Elimination half-life (day)	PrEP	15.89 (10.29, 21.50)	0.08
	Non-PrEP	17.53 (11.14, 23.92)	
	Difference	-1.63 (-3.44, 0.17)	
Steady state area under the curve [AUC[d] (day/mL)]	PrEP	1.73 (1.55, 1.90)	**<0.001**
	Non-PrEP	2.01 (1.81, 2.22)	
	Difference	-0.29 (-0.44, -0.13)	

PK: pharmacokinetics; PrEP: pre-exposure prophylaxis

[a] Covariate-adjusted mean by targeted minimum loss-based estimation (TMLE) (See Methods for more details).

[b] Confidence intervals (CIs) and p-values based on empirical variances estimated via the bootstrap procedure. **Bold**= significant

[c] P-values adjusted by the Holm method to control for family-wise error rate.

[d] Area under the time-concentration curves divided by dose amount.

Supplementary Table 5. Covariate-adjusted comparisons of pharmacokinetics features between non-PrEP users (n=24) and PrEP users who initiated PrEP after study enrollment (n=17) via TMLE. All comparisons were adjusted for baseline age, body weight, race, risk score, creatinine clearance, IFN-gamma, and IL-10 levels. All TMLE estimation results of means were averaged over 20 runs with a fixed random seed on top of the 10-fold cross-validation estimation procedure to ensure stability of the estimates. A bootstrap procedure based on 500 datasets was used to calculate the empirical variances of the estimates for each group and to derive the 95% confidence interval, as well as to test for a non-zero mean difference between the two groups via the Wald test. The Holm method was used to adjust for multiple comparisons of the five PK features.

**PK feature**	**PrEP User Status**	**Mean[a] (95% CI)[b]**	**2-sided raw p-value[b]**	**2-sided multiplicity-adjusted p-value[c]**
Clearance [CL (L/day)]	PrEP	0.58 (0.52, 0.64)	0.06	0.15
	Non-PrEP	0.53 (0.47, 0.60)		
	Difference	0.05 (0, 0.10)		
Volume of the peripheral compartment [Vp (L)]	PrEP	4.92 (2.14, 7.70)	0.25	0.32
	Non-PrEP	5.10 (2.37, 7.83)		
	Difference	-0.18 (-0.49, 0.13)		
Distribution half-life (day)	PrEP	2.34 (-3.40, 8.09)	0.87	0.87
	Non-PrEP	2.38 (-3.58, 8.34)		
	Difference	-0.04 (-0.49, 0.42)		
Elimination half-life (day)	PrEP	16.00 (10.13, 21.87)	0.11	0.18
	Non-PrEP	17.68 (11.15, 24.21)		
	Difference	-1.68(-3.73, 3.60)		
Steady state area under the curve [AUC[d] (day/mL)]	PrEP	1.80 (1.61, 1.98)	0.015	0.075
	Non-PrEP	1.99 (1.78, 2.20)		
	Difference	-0.20 (-0.35, -0.04)		

PK: pharmacokinetics; PrEP: pre-exposure prophylaxis

[a] Covariate-adjusted mean by targeted minimum loss-based estimation (TMLE) (See Methods for more details).

[b] Confidence intervals (CIs) and p-values based on empirical variances estimated via the bootstrap procedure. **Bold**= significant

[c] P-values adjusted by the Holm method to control for family-wise error rate.

[d] Area under the time-concentration curves divided by dose amount.

Supplementary Table 6. Estimates of PK parameters from the popPK model examining the effect of time-varying PrEP status on VRC01 clearance rate (CL). The popPK model is based on a two-compartment PK model parametrized in terms of CL, Vc, Q and Vp, with random effects for CL and Vp, and the effect of time-varying PrEP status on CL.

Parameter	Description	Estimate	95% CI	%RSE
**Fixed Effects**				
CL (L/day)	Clearance	0.52	(0.49, 0.56)	3.45
Vc (L)	Central volume	4.81	(4.75, 4.86)	0.58
Q (L/day)	Inter-compartmental clearance	0.56	(0.56, 0.57)	0.43
Vp (L)	Peripheral volume	4.94	(4.57, 5.31)	3.82
	PrEP effect on Ln(CL) (PrEP vs. non-PrEP)	0.02	(0.02, 0.03)	12.90
**Random Effects**				
CL (L/day)	SD, clearance	0.24	(0.19, 0.28)	10.51
Vp (L)	SD, peripheral volume	0.24	(0.18, 0.29)	12.04
**Error Model Parameters**				
Proportional error	SE	0.24	(0.23, 0.26)	2.40

RSE: relative standard error; CI: confidence interval; SE: standard error; SD: standard deviation

Supplementary Table 7. Estimates of PK parameters from the popPK model examining the effect of time-varying PrEP status on VRC01 clearance rate (CL), additionally adjusted for body weight. The popPK model is based on a two-compartment PK model parametrized in terms of CL, Vc, Q and Vp, with random effects for CL and Vp, and the effect of time-varying PrEP status and body weight on CL.

Parameter	Description	Estimate	95% CI	%RSE
**Fixed Effects**				
CL (L/day)	Clearance	0.53	(0.49, 0.56)	3.27
Vc (L)	Central volume	5.53	(5.46, 5.59)	0.60
Q (L/day)	Inter-compartmental clearance	0.51	(0.51, 0.52)	0.58
Vp (L)	Peripheral volume	4.59	(4.23, 4.96)	4.09
	Body weight effect on Ln(CL)	0.01	(0.00, 0.01)	42.66
	PrEP effect on Ln(CL) (PrEP vs. non-PrEP)	0.02	(0.02, 0.03)	7.00
**Random Effects**				
CL (L/day)	SD, clearance	0.22	(0.17, 0.27)	10.59
Vp (L)	SD, peripheral volume	0.25	(0.19, 0.32)	12.59
**Error Model Parameters**				
Proportional error	SE	0.24	(0.23, 0.26)	2.42

RSE: relative standard error; CI: confidence interval; SE: standard error; SD: standard deviation

Supplementary Table 8. Quantification of VRC01 serum concentrations not interfered by TFV/FTC based on the ELISA assay used for PK measurements of VRC01 in the study.

Sample with Spike-In	Sample Concentration (ug/mL)	Average OD over 2 technical replicates	Concentration % Recovery
Emtricitabine	100	2.05	115
	20	2.03	114
	4	2.04	115
	0.8	2.04	114
	0.16	2.13	122
	0	2.10	119
Tenofovir Disoproxil Fumarate	100	1.85	99
	20	1.72	89
	4	1.76	92
	0.8	1.75	92
	0.16	1.71	88
	0	1.81	96
Emtricitabine: Tenofovir Disoproxil Fumarate (2:3)	100	1.85	99
	20	1.84	98
	4	1.80	95
	0.8	1.89	103
	0.16	1.90	103
	0	1.87	101
VRC01 Spike in Assay Diluent	0	1.86	100

Supplementary References

1. Anderson PL, Liu AY, Castillo-Mancilla JR, et al. Intracellular Tenofovir-Diphosphate and Emtricitabine-Triphosphate in Dried Blood Spots following Directly Observed Therapy. *Antimicrob Agents Chemother*. Jan 2018;62(1)doi:10.1128/aac.01710-17
2. Zheng JH, Rower C, McAllister K, et al. Application of an intracellular assay for determination of tenofovir-diphosphate and emtricitabine-triphosphate from erythrocytes using dried blood spots. *J Pharm Biomed Anal*. Apr 15 2016;122:16-20. doi:10.1016/j.jpba.2016.01.038
3. Castillo-Mancilla JR, Zheng JH, Rower JE, et al. Tenofovir, emtricitabine, and tenofovir diphosphate in dried blood spots for determining recent and cumulative drug exposure. *AIDS Res Hum Retroviruses*. Feb 2013;29(2):384-90. doi:10.1089/aid.2012.0089
4. Niu X, Kubiak RW, Siriprakaisil O, et al. Tenofovir-Diphosphate in Dried Blood Spots vs Tenofovir in Urine/Plasma for Oral Preexposure Prophylaxis Adherence Monitoring. *Open Forum Infect Dis*. Aug 2022;9(8):ofac405. doi:10.1093/ofid/ofac405
5. Thurman AR, Schwartz JL, Cottrell ML, et al. Safety and Pharmacokinetics of a Tenofovir Alafenamide Fumarate-Emtricitabine based Oral Antiretroviral Regimen for Prevention of HIV Acquisition in Women: A Randomized Controlled Trial. *EClinicalMedicine*. Jun 2021;36:100893. doi:10.1016/j.eclinm.2021.100893
