# Supplementary material for: Adults on pre-exposure prophylaxis (tenofovir-emtricitabine) have faster clearance of anti-HIV monoclonal antibody VRC01
Source: Nat Commun. 2023 Nov 28;14:7813. doi: 10.1038/s41467-023-43399-5 (PMC10684488; doi:10.1038/s41467-023-43399-5)
Supplement: Supplementary file 3 — Reporting Summary [file 41467_2023_43399_MOESM3_ESM.pdf]

nature portfolio

Corresponding author(s): Yunda Huang

Last updated by author(s): Jun 9, 2023

Reporting Summary

Nature Portfolio wishes to improve the reproducibility of the work that we publish. This form provides structure for consistency and transparency in reporting. For further information on Nature Portfolio policies, see our Editorial Policies and the Editorial Policy Checklist.

Statistics

For all statistical analyses, confirm that the following items are present in the figure legend, table legend, main text, or Methods section.

n/a	Confirmed	
☐	☒	The exact sample size (*n*) for each experimental group/condition, given as a discrete number and unit of measurement
☐	☒	A statement on whether measurements were taken from distinct samples or whether the same sample was measured repeatedly
☐	☒	The statistical test(s) used AND whether they are one- or two-sided<br>*Only common tests should be described solely by name; describe more complex techniques in the Methods section.*
☐	☒	A description of all covariates tested
☐	☒	A description of any assumptions or corrections, such as tests of normality and adjustment for multiple comparisons
☐	☒	A full description of the statistical parameters including central tendency (e.g. means) or other basic estimates (e.g. regression coefficient) AND variation (e.g. standard deviation) or associated estimates of uncertainty (e.g. confidence intervals)
☐	☒	For null hypothesis testing, the test statistic (e.g. *F*, *t*, *r*) with confidence intervals, effect sizes, degrees of freedom and *P* value noted<br>*Give P values as exact values whenever suitable.*
☒	☐	For Bayesian analysis, information on the choice of priors and Markov chain Monte Carlo settings
☒	☐	For hierarchical and complex designs, identification of the appropriate level for tests and full reporting of outcomes
☐	☒	Estimates of effect sizes (e.g. Cohen's *d*, Pearson's *r*), indicating how they were calculated

Our web collection on statistics for biologists contains articles on many of the points above.

Software and code

Policy information about availability of computer code

Data collection	Clinical data were collected through Case Report Forms (CRFs) that are part of an electronic data capture (EDC) system or through electronicpatient-reported outcome (ePRO). Laboratory data were collected at respective research labs. Specifically, for Dry Blood Spot (DBS) data, LC-MS/MS analysis using a Thermo Scientific TSQ Vantage triple quadrupole mass spectrometer was used. For PK data, an ELISA with Bioplex software was used (Bioplex Manager, version 6.1). For Inflammatory marker data, the MSD panel and platform were used. For LBP and IFAB-P data, ELISA assay with SpectraMax i3X plate reader was used. For ADA data, the MSD panel and platform were used. See more details in Methods.
Data analysis	Code implementing methods, as well as all datasets will become publicly available upon acceptance of the manuscript. Instructions for installation and use will be given in the accompanying README file. During the review stage, a zip file containing code, accompany datasets and a README file is available to editors and reviewers.

For manuscripts utilizing custom algorithms or software that are central to the research but not yet described in published literature, software must be made available to editors and reviewers. We strongly encourage code deposition in a community repository (e.g. GitHub). See the Nature Portfolio guidelines for submitting code & software for further information.

Data

Policy information about availability of data

All manuscripts must include a data availability statement. This statement should provide the following information, where applicable:

- Accession codes, unique identifiers, or web links for publicly available datasets
- A description of any restrictions on data availability
- For clinical datasets or third party data, please ensure that the statement adheres to our policy

The data underlying the findings of this manuscript will be publicly available on figshare accession code:https://doi.org/10.6084/m9.figshare.23800698. All individual participant data have been de-identified.

Research involving human participants, their data, or biological material

Policy information about studies with human participants or human data. See also policy information about sex, gender (identity/presentation), and sexual orientation and race, ethnicity and racism.

Reporting on sex and gender	All participants in the randomized trial of data we sampled from were assigned male sex at birth and transgender persons who have sex with men. All but one of the participants we sampled were male sex assigned at birth.
Reporting on race, ethnicity, or other socially relevant groupings	We reported on Black/African American or other racial identities, given that VRC01 was isolated from an African American. We include age, BMI, weight, vital signs, safety labs, inflammatory markers, and intestinal permeability. We also include a behavioral risk score which assesses participant behavioral vulnerability to HIV. We adjusted for several pre-defined variables to ensure comparability between the groups.
Population characteristics	Sample population characteristics are included in Table 1.
Recruitment	Participant recruitment for HVTN 704/HPTN 085 is detailed in Edupuganti et al. 2021 JAIDS. For this study, we randomly sampled a total of 234 participants from US-based HVTN 704/HPTN 085 sites who reached the week 88 study visit (16 weeks after the 10th and last VRC01 infusion) HIV-1 negative and who did not permanently discontinue infusions during trial follow-up. Among these 234 participants, 77 did not self-report PrEP use or access the PrEP referral program, and 157 self-reported PrEP use. All available DBS samples collected at infusion visits of these self-reported PrEP users and self-reported non-PrEP users were measured and the results, one per visit, were included in the definition of PrEP users and non-PrEP users.
Ethics oversight	Central and site-specific institutional review boards and ethics committees reviewed and approved the initial protocol and each subsequent version.

Note that full information on the approval of the study protocol must also be provided in the manuscript.

Field-specific reporting

Please select the one below that is the best fit for your research. If you are not sure, read the appropriate sections before making your selection.

☒ Life sciences ☐ Behavioural & social sciences ☐ Ecological, evolutionary & environmental sciences

For a reference copy of the document with all sections, see nature.com/documents/nr-reporting-summary-flat.pdf

Life sciences study design

All studies must disclose on these points even when the disclosure is negative.

Sample size	We randomly sampled a total of 234 participants from US-based HVTN 704/HPTN 085 sites who reached the week 88 study visit (16 weeks after the 10th and last VRC01 infusion) HIV-1 negative and who did not permanently discontinue infusions during trial follow-up. All available DBS samples collected at infusion visits of these self-reported PrEP users and self-reported non-PrEP users were measured and the results, one per visit, were included in the definition of PrEP users and non-PrEP users. A participant was defined as a PrEP user if they met all of the following criteria during the study: 1) accessed the PrEP referral program on at least one occasion per self-report, 2) self-reported intermittent or continuous PrEP use, and 3) confirmation of ≥ 3 positive TDF-FTC detection tests out of DBS samples collected at infusion visits. A participant was defined as a non-PrEP user if they did not self-report any PrEP use and had no DBS samples tested positive for TDF. A total of 24 PrEP users out of 31 eligible PrEP users and 24 non-PrEP users out of 32 eligible non-PrEP users were sampled, with an equal split in the low (10 mg/kg) and high (30 mg/kg) VRC01 dose groups.
Data exclusions	We excluded participants who did not reach the week 88 study visit, acquired HIV, or discontinued VRC01 infusions.
Replication	We conducted TMLE analysis, which is an alternative to standard linear or nonlinear regression that can improve robustness and efficiency. All TMLE estimation results of means were averaged over 20 runs with a fixed random seed on top of the 10-fold cross-validation estimation procedure to ensure stability of the estimates.
Randomization	Not applicable, as this is a secondary analysis of HVTN 704/HPTN 085 data. See sample size above for details on our sample. For details on randomization for HVTN 704/HPTN 085 see Corey et al. 2021 NEJM.

Blinding	For BAMA measurements, nonlinear mixed effects models were used to analyze individual-level concentrations over time. Laboratory staff conducting assays were blinded to group allocation during data collection and analysis.

Reporting for specific materials, systems and methods

We require information from authors about some types of materials, experimental systems and methods used in many studies. Here, indicate whether each material, system or method listed is relevant to your study. If you are not sure if a list item applies to your research, read the appropriate section before selecting a response.

Materials & experimental systems

n/a	Involved in the study
☐	☒ Antibodies
☒	☐ Eukaryotic cell lines
☒	☐ Palaeontology and archaeology
☒	☐ Animals and other organisms
☐	☒ Clinical data
☒	☐ Dual use research of concern
☒	☐ Plants

Methods

n/a	Involved in the study
☒	☐ ChIP-seq
☒	☐ Flow cytometry
☒	☐ MRI-based neuroimaging

Antibodies

Antibodies used	The VRC01 drug product stock concentrations were prepared at Duke. Ch58 (Duke Protein Production Facility) (nicely et al., 2015 EBiomedicine; Pollara et al. 2014 J. Virol)
Validation	The VRC01 drug product was used as a positive control; heat inactivation did not affect the neutralization activity of the VRC01 drug product when spiked into a normal human serum sample. The VRC01 drug product was assayed against each HIV-1 Env-pseudotyped virus three times at starting concentrations of 100 mcg/mL and 5 mcg/mL using eight 3-fold serial dilutions in duplicate. HIV-1 PVO.4 was included in each assay as a positive control to confirm the integrity of the VRC01 drug product. The assay has been formally validated for accuracy, sensitivity, specificity, precision, linearity, range and robustness.

Clinical data

Policy information about clinical studies

All manuscripts should comply with the ICMJE guidelines for publication of clinical research and a completed CONSORT checklist must be included with all submissions.

Clinical trial registration	NCT02716675
Study protocol	https://clinicaltrials.gov/ct2/show/NCT02716675
Data collection	This study is a secondary analysis utilizing data and samples collected in the study listed above. For details on data collection for HVTN 704/HPTN 085 see Corey et al. 2021 NEJM.
Outcomes	For outcome information for HVTN 704/HPTN 085 see Corey et al. 2021 NEJM.
